# Clinical evaluation of an interoperable clinical decision-support system for the detection of systemic inflammatory response syndrome in critically ill children

**DOI:** 10.1186/s12911-021-01428-7

**Published:** 2021-02-18

**Authors:** Antje Wulff, Sara Montag, Nicole Rübsamen, Friederike Dziuba, Michael Marschollek, Philipp Beerbaum, André Karch, Thomas Jack

**Affiliations:** 1 Peter L. Reichertz Institute for Medical Informatics of TU Braunschweig and Hannover Medical School, Karl-Wiechert-Allee 3, 30625 Hannover, Germany; 2 Institute of Epidemiology and Social Medicine, University of Muenster, Domagkstr. 3, 48149 Muenster, Germany; 3 Department of Pediatric Cardiology and Intensive Care Medicine, Hannover Medical School, Carl-Neuberg-Str. 1, 30625 Hannover, Germany

**Keywords:** Clinical decision support systems, Diagnostic study, Pediatric intensive care units, Systemic inflammatory response syndrome

## Abstract

**Background:**

Systemic inflammatory response syndrome (SIRS) is defined as a non-specific inflammatory process in the absence of infection. SIRS increases susceptibility for organ dysfunction, and frequently affects the clinical outcome of affected patients. We evaluated a knowledge-based, interoperable clinical decision-support system (CDSS) for SIRS detection on a pediatric intensive care unit (PICU).

**Methods:**

The CDSS developed retrieves routine data, previously transformed into an interoperable format, by using model-based queries and guideline- and knowledge-based rules. We evaluated the CDSS in a prospective diagnostic study from 08/2018–03/2019. 168 patients from a pediatric intensive care unit of a tertiary university hospital, aged 0 to 18 years, were assessed for SIRS by the CDSS and by physicians during clinical routine. Sensitivity and specificity (when compared to the reference standard) with 95% Wald confidence intervals (CI) were estimated on the level of patients and patient-days.

**Results:**

Sensitivity and specificity was 91.7% (95% CI 85.5–95.4%) and 54.1% (95% CI 45.4–62.5%) on patient level, and 97.5% (95% CI 95.1–98.7%) and 91.5% (95% CI 89.3–93.3%) on the level of patient-days. Physicians’ SIRS recognition during clinical routine was considerably less accurate (sensitivity of 62.0% (95% CI 56.8–66.9%)/specificity of 83.3% (95% CI 80.4–85.9%)) when measurd on the level of patient-days. Evaluation revealed valuable insights for the general design of the CDSS as well as specific rule modifications. Despite a lower than expected specificity, diagnostic accuracy was higher than the one in daily routine ratings, thus, demonstrating high potentials of using our CDSS to help to detect SIRS in clinical routine.

**Conclusions:**

We successfully evaluated an interoperable CDSS for SIRS detection in PICU. Our study demonstrated the general feasibility and potentials of the implemented algorithms but also some limitations. In the next step, the CDSS will be optimized to overcome these limitations and will be evaluated in a multi-center study.

*Trial registration*: NCT03661450 (ClinicalTrials.gov); registered September 7, 2018.

**Supplementary Information:**

The online version contains supplementary material available at 10.1186/s12911-021-01428-7.

## Background

Sepsis, an imbalance between pro- and anti-inflammation as the body’s response to an infectious agent [1], is one of the most common and critical conditions entailing high morbidity and mortality in critically ill children [2–6]. Specific age-dependent definitions have been provided by the International Pediatric Sepsis Consensus Conference (IPSCC) in 2005 [7]; in addition to evidence for an infectious agent, these definitions require the presence of a systemic inflammatory response syndrome (SIRS). Although the newest Sepsis-3 guidelines for adults removed this relationship between SIRS and sepsis [8, 9], the definitions are still valid for children due to a different clinical course in younger patients. SIRS in pediatric patients may quickly proceed to severe sepsis, septic shock and multiple organ failure [10]. In pediatric cardiothoracic patients, SIRS was related to a prolonged stay in pediatric intensive care (PICU) with all entailed risks [11]. Early recognition of pediatric SIRS is important for a timely commencement of treatment and sepsis diagnostics.

Digitalization in healthcare has fostered the development of clinical decision-support systems (CDSS) capable of supporting human decision-making by reusing routinely documented data [12, 13]. However, current research for pediatric SIRS detection by CDSS is scarce [14]. Related approaches were described by Dewan et al. [15], Scott et al. [16], Vidrine et al. [17], Le et al. [18], Sepanski et al. [19], Cruz et al. [20] and Eisenberg et al. [21], but focused on severe sepsis, septic shock, or therapy improvements rather than SIRS diagnosis. To our knowledge, a CDSS for detection of pediatric SIRS has not yet been successfully developed. Furthermore, related CDSS were only rarely tested under clinical routine settings as neither routine data nor appropriate reference standards were used [14]. We designed a knowledge-based CDSS for pediatric SIRS detection that uses routine data from a patient data management system (PDMS) and implements algorithms based on guidelines and experts’ knowledge assets [22]. Our CDSS is based on an interoperability standard for clinical information modelling (*openEHR* [23]), international terminologies and model-based, standardized data queries, to overcome the CDSS dependence to local infrastructures and to facilitate cross-institutional reuse.

In this article, we present the results of a thoroughly performed diagnostic study for evaluating the diagnostic accuracy of our CDSS using clinical monitoring data, previously transformed into standardized data formats, computerized experts’ knowledge and international guidelines for SIRS detection in critically ill children.

## Methods

### Study design

The study is reported in accordance with the Standards for Reporting of Diagnostic Accuracy Studies (STARD) (see Additional file 1: Appendix 1) [24]. The study protocol has been approved by the Ethics Committee of Hannover Medical School and published [25].

This diagnostic study was designed to evaluate CDSS accuracy by using the reference standard defined by two experienced clinicians on the base of IPSCC SIRS criteria (*primary aim*). The *secondary aim* was to compare CDSS accuracy to the accuracy of assessments of clinicians working in clinical routine to assess SIRS awareness of clinicians during challenging routine work [25]. The study took place at the PICU of Hannover Medical School. Sensitivity and specificity on the level of patients (= a patient’s PICU stay) and on the level of patient-days (= intensive care days) were defined as *primary* and *secondary outcome measure*, respectively. The patient level analysis summarizes the analysis on the level of patient-days in a conservative way so that e. g. individuals with SIRS can also contribute to the estimation of specificity (see below). Sensitivity (alternative hypothesis: 98%, null hypothesis: 90%) and specificity (alternative hypothesis: 90%, null hypothesis: 80%) were chosen as the *co-primary endpoint* [25]. A sample size of 97 patients with at least one SIRS episode, and 137 patients with or without a SIRS episode was calculated based on these assumptions (type I error = 0.05, power of 90%; chi square test) [25]. Details of three subsequent changes to the protocol are given in the Additional file 1: Appendix 2.

### Participants

Recruitment started in August 2018 and ended in March 2019. PICU patients were eligible if (1) aged between 0 and 18 years, (2) an informed consent was obtained, (3) the length of stay exceeded 12 h and (4) standard clinical data monitoring in the PDMS was carried out. Patients were treated according to standard of care.

### Test methods

The self-developed CDSS is an application that is based on an open data platform, in which various data sets from different primary source systems are gathered together in a standardized, unambiguous format by using a semantic interoperability standard for representation of clinical information called *openEHR* [23]. By this, and in contrast to recent stand-alone, institution-specific and locked-in solutions, our CDSS will be easily shareable with other institutions following the same standard because a shared meaning of data that will be used by the CDSS is formed (semantic interoperability). Furthermore, this prevents that incorrect results of the CDSS occur because wrongly interpreted data are inserted into the algorithm.

After recruitment finished, the required routine data were integrated from the PDMS of the intensive care unit into this standardized data repository, based on internationally agreed-upon data models (*openEHR archetypes*) and terminologies (e. g. *LOINC*). The data used in the CDSS comprise demographic data (e. g. date of birth), vital signs (e. g. body temperature, respiratory rate, heart rate), laboratory values (e. g. leucocyte count), procedures and medical devices (e. g. pacemaker, cooling devices). All data items integrated in the standardized data platform and used for CDSS assessment can be found in Additional file 1: Appendix 3. For details of the CDSS, we refer to Wulff et al. [22]. The CDSS implements model-based data queries by using the openEHR Archetype Query Language (AQL) to retrieve these data sets in an unambiguous format. The CDSS consists of a knowledge base comprising a working memory and a rule base. The routine data sets retrieved are inserted as dynamic facts into the working memory. The rule base includes all rules related to SIRS diagnosis which were derived from the international SIRS criteria for children by the IPSCC [7]. Here, pediatric SIRS is defined as the presence of at least two out of four criteria (abnormal body temperature, leucocyte count, heart rate, respiratory rate based on age-specific norm values), one of which must be an abnormal body temperature or leukocyte count [7].

Based on the standardized, semantically-enriched routine patient data and these algorithms, the CDSS started to operate by deciding on the presence or absence of SIRS episodes (*diagnostic approach I*) [22].

In parallel to patient recruitment, clinicians performed a real-time SIRS assessment by filling in pseudonymized digital forms per shift without chart review (*diagnostic approach II*).

Two experienced pediatricians defined the *reference standard* by blinded, retrospective digital chart review and analysis based on the above mentioned IPSCC pediatric SIRS criteria. In case of disagreement, a third clinician was consulted. A day was defined as *SIRS-positive,* if the patient suffered from SIRS for at least one full hour per day. The starting time of the *SIRS episode* was marked, and the end was documented as soon as SIRS criteria were not fulfilled for a minimum of 24 h.

### Data preparation

Results were assessed *per patient’s PICU stay* according to six cases (1) false positive, (2) true positive, (3) false negative, (4) true negative, (5) false negative and false positive, (6) false positive and true positive. Every SIRS episode needed to be detected within −/+ 4 h of the episode starting time according to the reference standard documentation [25] (see Additional file 1: Appendix 4). Results were also assessed *per intensive care day* according to the first four cases.

### Data analysis

Diagnostic accuracy on the level of a patient’s PICU stay was used as *primary outcome measure*. Additionally, the specificity among patients who had no SIRS during their stay was estimated to assess the probability of false alarms among unaffected patients. Diagnostic accuracy on the level of intensive care days was used as *secondary outcome measure*. Sensitivity and specificity with Wald 95% CI were estimated via Generalized Estimating Equations (GEE) [26] using R version 4.0.2 [27] and R package *geepack* (version 1.3–1) [28]. Subgroup analyses were conducted among patients younger and older than 12 months.

Missing values in the *diagnostic approach II* were excluded for the primary analysis (complete case analysis). To quantify the impact of missingness, we calculated sensitivity and specificity under the assumption that all missing days were either rated correctly (i.e., imputation as true positive or true negative) or incorrectly (i.e., imputation as false positive or false negative).

## Results

### Participants

**Fig. 1:**
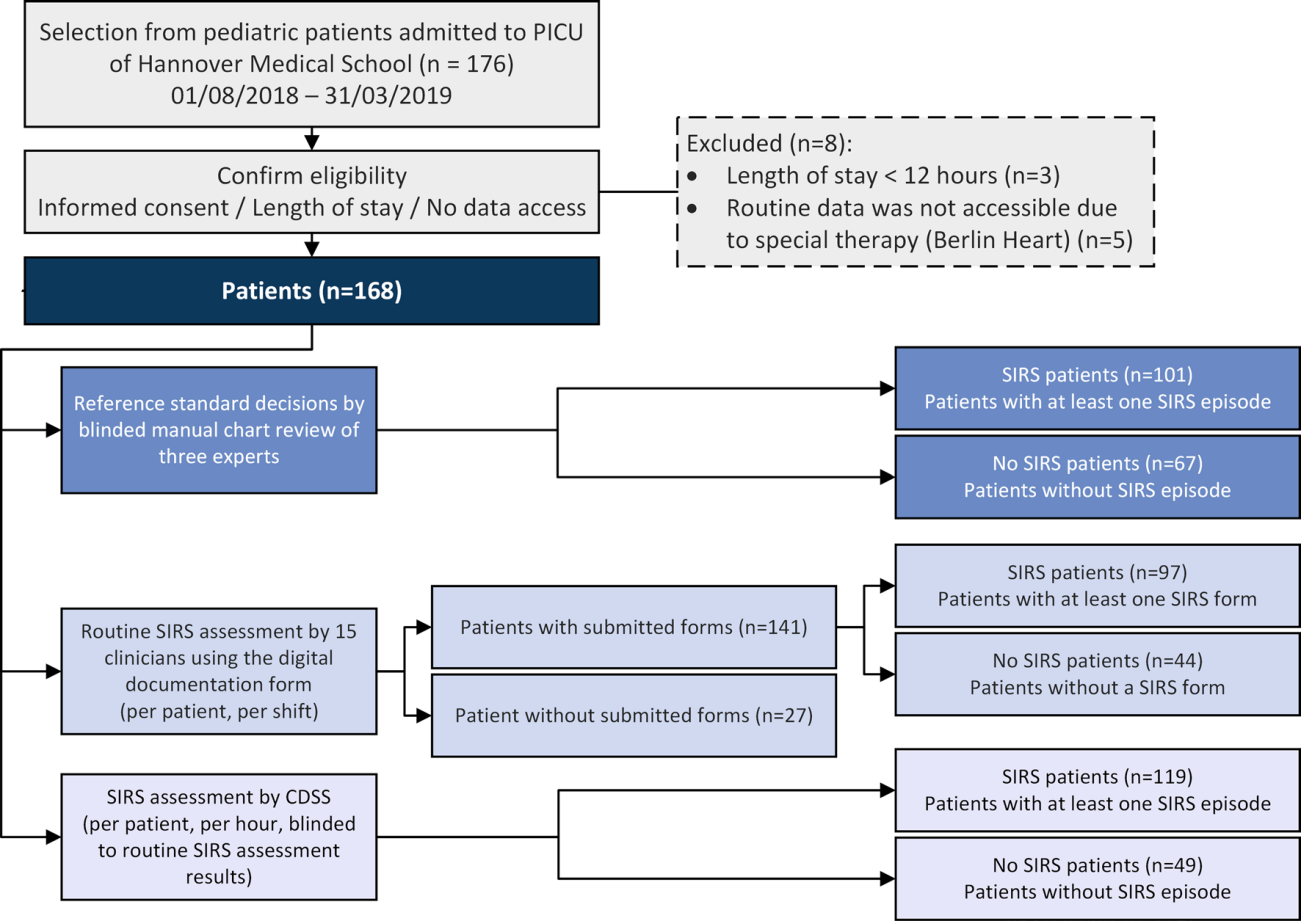
Flow diagram for recruited patients (PICU; pediatric intensive care unit)

Recruitment resulted in a final effective sample size of n = 168 (with 1,998 days), with 101 *SIRS* patients (60.1%) and 67 *No SIRS* patients (39.9%, Fig. 1). This fulfilled the pre-required sample size of 97 patients with at least one SIRS episode, and 137 patients with or without a SIRS episode was required [25]. Overall, the patients experienced 210 *SIRS episodes* (see enhanced flow diagram with intensive care days and stays in Additional file 1: Appendix 5) with 123 alerts for abnormal respiratory rate, 39 for heart rate, 58 for temperature, 117 for lowered/elevated leucocyte count or left shift of neutrophils.

Baseline characteristics of the patients are shown in Table 1. The mean length of an intensive care stay was 12 days; 42 of 168 patients (25.0%) had multiple stays.^1^ Overall PICU mortality was 4.8% (8/168).

**Table 1:** Baseline characteristics of participants (n = 168)

Patient characteristics	Patients n = 168 (%)
Age*	
0 days to 1 week	18 (11%)
1 week to 1 month	10 (6%)
1 month to 1 year	60 (36%)
2–5 years	44 (26%)
6–12 years	25 (15%)
13 to < 18 years	11 (7%)
Gender	
Female	64 (38%)
Male	104 (62%)
Underlying disease category	
*Surgical*	
Cardiology (with cardiopulmonary bypass)	78 (46%)
Pediatric surgery	9 (5%)
*Non-surgical*	
Cardiology	20 (12%)
Oncology	4 (2%)
Metabolic disease	3 (2%)
*Mixed*	
Pulmonology (including lung transplantation)	17 (10%)
Gastroenterology (including liver transplantation)	16 (10%)
Neurology/neurosurgery	11 (7%)
Others (ear-nose and throat, immunology, maxillofacial surgery, trauma surgery)	9 (5%)
Nephrology (including kidney transplantation)	1 (1%)
Mortality	8 (5%)

*Median age in years (range) = 2 (0–17)

## Test results

### Diagnostic approach I (CDSS assessment)

**Fig. 2:**
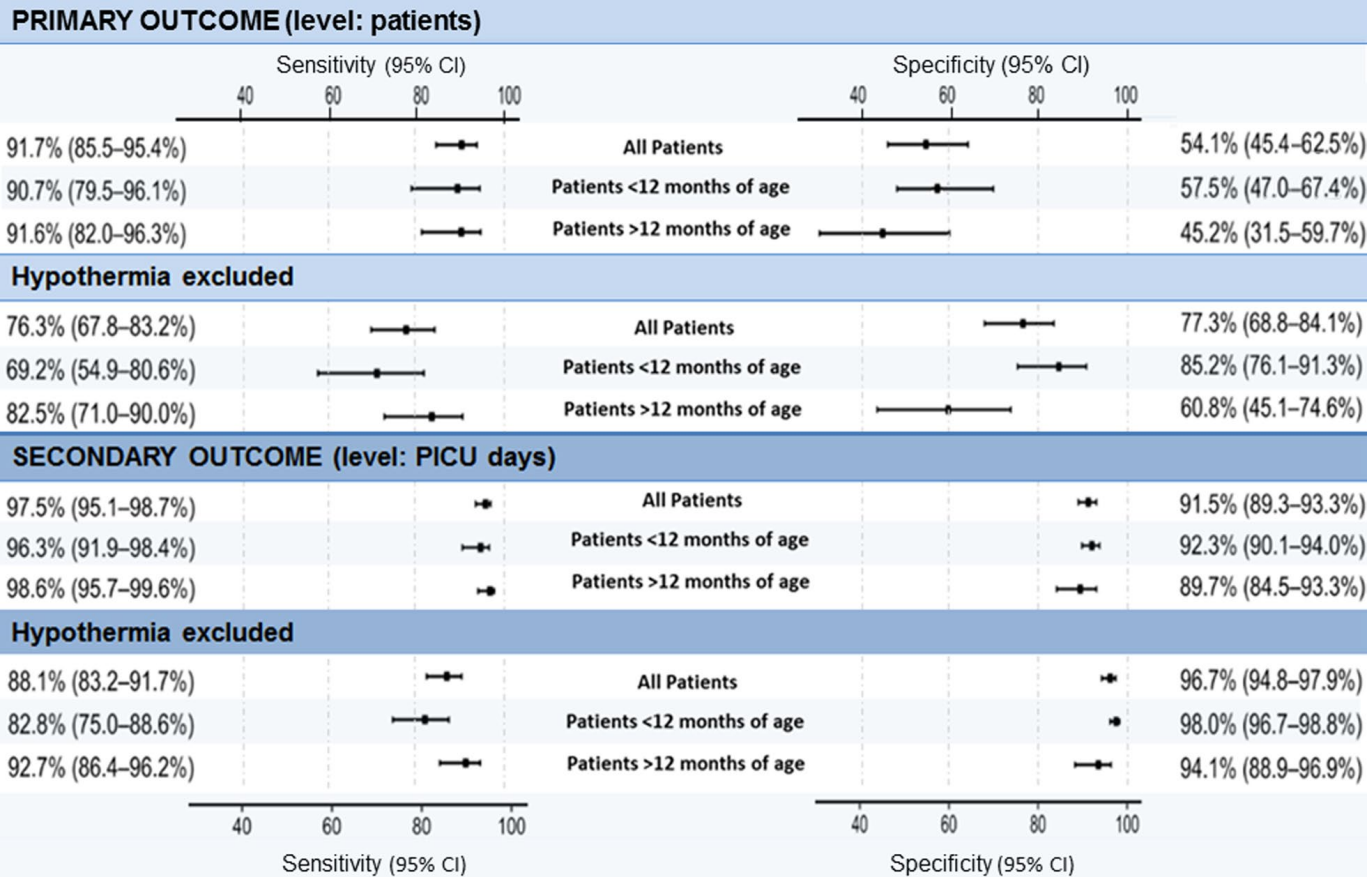
Summarized results of the CDSS regarding primary and secondary outcome criteria (including results when hypothermia rules are excluded)

On the level of patients (Table 2), sensitivity was 91.7% (95% CI 85.5–95.4%) and specificity was 54.1% (95% CI 45.4–62.5%). Among patients, who had no SIRS according to the reference standard, specificity was 73.0% (95% CI 63.2–81%). Comparison of the lower bound of the 95% confidence interval with the predefined null hypothesis in the primary endpoints (sensitivity of 90% and specificity of 80%) [25], revealed that we were not able to reject it. When stratifying by age, specificity was higher among children younger than 12 months but lower among older children while sensitivity did not vary (Fig. 2).

**Table 2:** Contingency table for evaluating the accuracy of the CDSS *on the level of patients*

CDSS	Reference standard		
	Positive	Negative	Total
Positive	80 + 32^1^	27 + 2 + 32^3^	173
Negative	8 + 2^2^	79	89
Total	122	140	262^4^

^1^True positive (case 2) + false positive/true positive (case 6)

^2^False negative (case 3) + false positive/false negative (case 5)

^3^False positive (case 1) + false positive/false negative (case 5) + false positive/true positive (case 6)

^4^228 patient’s stays (PICU stay) were assessed

Because hypothermia was under discussion for being a non-valid criterion before, a sensitivity analysis was performed excluding the hypothermia rule from the CDSS algorithm. When hypothermia was not included, specificity was higher but sensitivity was lower. Among patients who had no SIRS according to the reference standard, specificity was 94.2% (95% CI 87.5–97.4%). Exclusion of hypothermia increased specificity among children younger than 12 months but decreased it among older children.

On the level of intensive care days (Table 3), sensitivity was 97.5% (95% CI 95.1–98.7%) and specificity was 91.5% (95% CI 89.3–93.3%). Specificity was higher among children younger 12 months, but lower among older children. Exclusion of hypothermia in the CDSS SIRS definition resulted in a higher specificity but a lower sensitivity.

**Table 3:** Contingency table for evaluating the accuracy of the CDSS *on the level of intensive care days*

CDSS	Reference standard		
	Positive	Negative	Total
Positive	450	143	593
Negative	10	1395	1405
Total	460	1538	1998

### Diagnostic approach II (Routine assessment)

The clinicians submitted 1,704 forms for 141 patients. No forms were available for 27 patients (Fig. 1). 563 additional forms were available but 32 were submitted outside the selected PICU stay of the recruited patient and 531 could not be assigned to a patient. On average, 12 forms per patient, 14 forms per day and 219 forms per clinician were submitted. With increasing study duration, the compliance for documentation decreased (Additional file 1: Appendix 6). Consequently, assessments for 725 out of 1,998 days were missing. Missingness was independent of SIRS status (36.6% of data were missing of 462 days with SIRS; 36.2% of data were missing of 1,536 days without SIRS). In the complete case analysis, sensitivity was 38.1% (95% CI 32.5–44%) and specificity was 71.5% (95% CI 68.6–74.3%). If we assume that all 725 days with missing routine assessments would have been rated correctly evaluated by the routine assessors (true positive or true negative), sensitivity would be 62.0% (95% CI 56.8–66.9%) with a specificity of 83.3% (95% CI 80.4–85.9%). If all missing days would have been rated incorrect, sensitivity would be 23.3% (95% CI 18.8–28.5%) and specificity 39.7% (95% CI 35.9–43.6%).

## Discussion

SIRS plays a key role in the development of organ dysfunction in critically ill children and determine morbidity and mortality [11, 29–31]. Therefore, in this study, we evaluated a self-developed interoperable CDSS for detection of SIRS in pediatric patients. By supporting the diagnosis of SIRS, the CDSS is able to detect one of the earliest signs for clinical deterioration. By this, early treatment can be initiated and progression to severe SIRS or sepsis, organ failure and death might be preventable. Proving this effect and the clinical benefit of the implementation of the CDSS will be part of further investigations in a randomized interventional study, as we have decided to demonstrate the diagnostic accuracy of the method in this first step.

While the CDSS did not reach the pre-defined primary endpoints of 90% sensitivity and 80% specificity on a patient level (which is a very conservative approach to estimate the diagnostic accuracy of the CDSS), the diagnostic accuracy on the level of patient-days was much higher than the one of physicians’ real-time ratings. Unfortunately, routine SIRS assessment had a low compliance with some assessments missing. However, even in the best case scenario, where all missing days were imputed as correctly diagnosed, the sensitivity on patient-day level was only 62.0%. This illustrates the potentials for implementing a CDSS in this setting. In particular, less experienced clinicians could be supported by the CDSS, acting as a *co-pilot* [32], because they often do not suspect SIRS and, thus, miss early initiation of sepsis treatment and diagnostics.

However, CDSS development is still in progress, since this study also showed weaknesses, which will be optimized in future work (all misclassifications are summarized in Additional file 1: Appendix 7). All errors by the algorithm itself were caused by a wrong interpretation of a dependence between respiratory rate and mechanical ventilation, so that this specific rule will be modified. Furthermore, new data sets will be integrated because patients often suffered from underlying diseases, undergoing procedures or taking drugs that caused hypothermia, elevated or lowered heart rate, or leukocytosis, thus not interpretable as SIRS sign.

Although intensive care environments are often characterized by a high-quality technical infrastructure with continuous data monitoring, another reason for errors was a low data quality due to either inconclusive values, that have been manually validated, or missing values. Furthermore, false positive alerts were often caused by borderline values for the IPSCC criteria. Since the quality of primary source data might differ between institutions, the accuracy of the CDSS also might vary. These aspects will be examined in detail by testing more flexible approaches (e. g. fuzzy logic) and evaluating the CDSS in a multi-center study.

Most of the misclassifications can be overcome by incorporating additional algorithms and variables. However, errors that occurred because individual patient situations need to be rated other than defined by guidelines, will be difficult to overcome by conventional knowledge-based approaches. One example is hypothermia, which had a relevant impact on specificity. Many factors associated with a patient’s individual situation are influencing temperature and temperature measurement. Our study showed that ignoring hypothermia as a SIRS criterion is not helpful, because this considerably decreases sensitivity. Since hypothermia is prone to errors especially in children > 12 months, a rule adaption might lead to increased sensitivity and specificity. The incorporation of machine learning algorithms able to determine a patient’s individual baseline or to learn new relations in real-time might be valuable, too.

The development and evaluation of an interoperable CDSS for SIRS detection was a first step in the process. Currently, we are working on the integration of microbiological results and started to include the IPSCC criteria for organ dysfunction and failure. The combination of the CDSS with a prediction model for the differentiation between SIRS and sepsis, e. g. as published by our study group [33], could raise additional benefits to our approach. Furthermore, since all experts in our study come from the same department, this could have affected the reference standard, so that a further validation by using experts from different locations is aspired.

Due to our interoperable design, the reasoning procedures and knowledge base of our CDSS are functionally independent of the underlying local infrastructures. All queries used for retrieving data needed in the CDSS can be shared with other institutions without modifications as long as the same (inter)national openEHR data models are in use [22]. We already tested this approach with a prototypical application for outbreak detection of pathogens in hospitals and tracking of COVID-19 patients. This tool was built upon the architectural idea of the CDSS of this work and was successfully rolled out quickly to other university medical centers which integrated their primary source data using the same standard for data representation and terminologies [34, 35]. Therefore, we expect that an implementation of our CDSS for SIRS detection at another institution which follows the same interoperability approach will be possible, too. Afterwards, the conduction of a multi-center study with an optimized CDSS will be another future step. Further evaluations will also encompass real-time performance of the CDSS and clinical relevance about improvement of goal-directed therapy concerning SIRS and sepsis.

We are aware that our approach of determining CDSS accuracy by comparison with decisions made by manual chart review comes with weaknesses in terms of objectivity. However, it is still one of the best approaches to reach a reference standard that fits the type of decisions made in clinical settings. Alternatives, such as ICD codes, are inaccurate in terms of sensitivity or timing. Nevertheless, it is a time-consuming approach demonstrating the impossibility of manually assessing large retrospective datasets as needed for developing machine learning algorithms. A knowledge-based CDSS, such as the one presented in this study, might be a tool to reliably label large retrospective data sets to make them available for machine learning training purposes. This is of particular interest, because SIRS-labeled training data for pediatric patients are currently not available [14].

To our knowledge, we present the first interoperable CDSS for detection of pediatric SIRS that has been successfully evaluated in a clinical-driven study, using routine data, broad eligibility criteria for patients of all pediatric ages and underlying diseases, and an appropriate reference standard. Previous CDSS rather tried to optimize SIRS criteria or were using non-specific sepsis criteria, often with impressive results [15, 19, 20]; other approaches aimed at predicting severe sepsis [16, 18] or improving time to goal-directed therapy [17]. All these studies focused on recognizing severe sepsis or septic shock directly instead of SIRS as the initial clinical feature; some used their own criteria differing from the IPSCC definition or set different age ranges, excluding newborns, infants or young adults, thereby limiting the routine (re)use of such system [14]. Often, the reference standard used seems problematic such as in Dewan et al. [15], who chose initiated treatment as reference. The documented time of treatment might not reflect the clinically relevant time as SIRS onset is often missed during clinical routine, as underlined by our findings.

## Conclusions

We successfully evaluated a self-developed, interoperable CDSS for SIRS detection in pediatric patients ranging from newborns to young adults. The CDSS is based on an interoperable concept facilitating the reuse of the CDSS across institutions.

Our study results demonstrated the general feasibility of the implemented algorithms while specificity on patient level was not as good as expected. Several strategies will be combined to minimize the false positive alerts and optimize the CDSS before conducting a multi-center study. Nevertheless, the low diagnostic accuracy results of the routine assessment show that awareness for SIRS seems quite low, thus, underlining that this clinical domain is in need for CDSS implementation.

## Supplementary Information

**Additional file 1:** **Appendix 1**. STARD checklist. **Appendix 2**. Details of changes to published study protocol.** Appendix 3**. Routine data used from patient data management system for CDSS assessment of all participants.** Appendix 4**. Strategy for SIRS episode evaluation with examples.** Appendix 5**. Flow diagram for recruited patients with intensive care days and patient’s PICU stays.** Appendix 6**. Submitted forms during routine assessment per shift and per day.** Appendix 7**. False decisions from the CDSS diagnostic approach, classified into error categories.

## Data Availability

The patients’ datasets generated and analyzed during the current study are not publicly available due to data protection and security reasons but are available in a pseudonymized format from the corresponding author upon reasonable request and with permission of the data security officer of Hannover Medical School. All data models used for the developed CDSS can be found at https://ckm.highmed.org/ckm. All information on the design of the developed CDSS can be found in the article by Wulff et al. [22], which was previously published open access. The CDSS prototype is available from the corresponding author upon reasonable request and with the permission of the further developers.

## Abbreviations

CDSS: clinical decision-support system
CI: confidence interval
ECMO: extracorporeal membrane oxygenation
GEE: generalized estimating equations
IPSCC: international pediatric sepsis consensus conference
PICU: pediatric intensive care unit
PDMS: patient data management system
SIRS: systemic inflammatory response syndrome
STARD: standards for reporting of diagnostic accuracy studies

## Footnotes

1 28 patients with 2 stays, 11 patients with 3 stays, 2 patients with 4 stays and 1 patient with 5 stays.

## Original protocol

The original protocol of the study has been made available by publication (BMJ Open 2019, https://doi.org/10.1136/bmjopen-2019-028953).

## Statistical review

The analysis of the diagnostic study was conducted by NR and reviewed by AK at the Institute of Epidemiology and Social Medicine, University of Muenster.
