# Supplementary material for: Clinical evaluation of an interoperable clinical decision-support system for the detection of systemic inflammatory response syndrome in critically ill children
Source: BMC Med Inform Decis Mak. 2021 Feb 18;21:62. doi: 10.1186/s12911-021-01428-7 (PMC7889709; doi:10.1186/s12911-021-01428-7)
Supplement: Supplementary file 1 — Additional file 1: Appendix 1. STARD checklist. Appendix 2. Details of changes to published study protocol. Appendix 3. Routine data used from patient data management system for CDSS assessment of all participants. Appendix 4. Strategy for SIRS episode evaluation with examples. Appendix 5. Flow diagram for recruited patients with intensive care days and patient’s PICU stays. Appendix 6. Submitted forms during routine assessment per shift and per day. Appendix 7. False decisions from the CDSS diagnostic approach, classified into error categories. [file 12911_2021_1428_MOESM1_ESM.pdf]

Clinical Evaluation of an Interoperable Clinical Decision-Support System for the Detection of Systemic Inflammatory Response Syndrome in Critically Ill Children

Antje Wulff, M.Sc.[a,1*], Sara Montag, MD[a,1*], Nicole Rübsamen, PhD[b], Friederike Dziuba, Dr. med.[c], Michael Marschollek, Prof.[a], Philipp Beerbaum, Prof.[c], André Karch, Prof.[b], Thomas Jack, Dr. med.[c]

[a]*Peter L. Reichertz Institute for Medical Informatics of TU Braunschweig and Hannover Medical School, Karl-Wiechert-Allee 3, 30625 Hannover, Germany*

[b]*Institute of Epidemiology and Social Medicine, University of Muenster, Domagkstr. 3, 48149 Muenster, Germany*

[c]*Department of Pediatric Cardiology and Intensive Care Medicine, Hannover Medical School, Carl-Neuberg-Str. 1, 30625 Hannover, Germany*

*these authors *contributed equally* to the work

[1]Corresponding authors at:
Peter L. Reichertz Institute for Medical Informatics of
TU Braunschweig and Hannover Medical School,
Karl-Wiechert-Allee 3, 30625 Hannover, Germany.
E-mail address: antje.wulff@plri.de (A. Wulff), **Phone:** +49 (0) 531 / 391 – 9510, **ORCID:** 0000-0002-2550-2627
E-mail address: sara.montag@elisabethgruppe.de (S. Montag)

Email addresses for all authors:
antje.wulff@plri.de
sara.montag@elisabethgruppe.de
nicole.ruebsamen@ukmuenster.de
dziuba.friederike@mh-hannover.de
michael.marschollek@plri.de
beerbaum.philipp@mh-hannover.de
andre.karch@ukmuenster.de
jack.thomas@mh-hannover.de

ADDITIONAL FILE 1

Appendix 1: STARD checklist

Section & Topic	No	Item	Reported on page #
**TITLE OR ABSTRACT**			
	**1**	Identification as a study of diagnostic accuracy using at least one measure of accuracy<br>(such as sensitivity, specificity, predictive values, or AUC)	1,2
**ABSTRACT**			
	**2**	Structured summary of study design, methods, results, and conclusions<br>(for specific guidance, see STARD for Abstracts)	2
**INTRODUCTION**			
	**3**	Scientific and clinical background, including the intended use and clinical role of the index test	3,4
	**4**	Study objectives and hypotheses	3,4
**METHODS**			
Study design	**5**	Whether data collection was planned before the index test and reference standard were performed (prospective study) or after (retrospective study)	4
Participants	**6**	Eligibility criteria	5 + appendix 1
	**7**	On what basis potentially eligible participants were identified<br>(such as symptoms, results from previous tests, inclusion in registry)	5 + appendix 1
	**8**	Where and when potentially eligible participants were identified (setting, location and dates)	5 + appendix 1
	**9**	Whether participants formed a consecutive, random or convenience series	5 + appendix 1
Test methods	**10a**	Index test, in sufficient detail to allow replication	5,6
	**10b**	Reference standard, in sufficient detail to allow replication	6 (lines 181-186)
	**11**	Rationale for choosing the reference standard (if alternatives exist)	6,14,15
	**12a**	Definition of and rationale for test positivity cut-offs or result categories<br>of the index test, distinguishing pre-specified from exploratory	5,6
	**12b**	Definition of and rationale for test positivity cut-offs or result categories<br>of the reference standard, distinguishing pre-specified from exploratory	6
	**13a**	Whether clinical information and reference standard results were available<br>to the performers/readers of the index test	5,6
	**13b**	Whether clinical information and index test results were available<br>to the assessors of the reference standard	6
Analysis	**14**	Methods for estimating or comparing measures of diagnostic accuracy	6,7
	**15**	How indeterminate index test or reference standard results were handled	7
	**16**	How missing data on the index test and reference standard were handled	7
	**17**	Any analyses of variability in diagnostic accuracy, distinguishing pre-specified from exploratory	7
	**18**	Intended sample size and how it was determined	7
**RESULTS**			
Participants	**19**	Flow of participants, using a diagram	9 + appendix 3
	**20**	Baseline demographic and clinical characteristics of participants	8
	**21a**	Distribution of severity of disease in those with the target condition	N/A
	**21b**	Distribution of alternative diagnoses in those without the target condition	N/A
	**22**	Time interval and any clinical interventions between index test and reference standard	5,6, N/A (no clinical

			interventions)
Test results	**23**	Cross tabulation of the index test results (or their distribution) by the results of the reference standard	10,11
	**24**	Estimates of diagnostic accuracy and their precision (such as 95% confidence intervals)	9-12
	**25**	Any adverse events from performing the index test or the reference standard	N/A (no adverse events)
**DISCUSSION**			
	**26**	Study limitations, including sources of potential bias, statistical uncertainty, and generalisability	12-15
	**27**	Implications for practice, including the intended use and clinical role of the index test	12-15
**OTHER INFORMATION**			
	**28**	Registration number and name of registry	2
	**29**	Where the full study protocol can be accessed	4
	**30**	Sources of funding and other support; role of funders	23

Appendix 2: Details of changes to the published study protocol

The study protocol has been previously published. For the conduction of the study, it was modified in three points:

- Extension of exclusion criteria: Five recruited patients could not undergo standard data monitoring due to a special treatment (e. g. Berlin Heart*) and were therefore excluded.
- Definition of a shift: Correct detection of a SIRS episode was originally defined as S*IRS identified within the correct shif*t. This would mean that a SIRS episode starting shortly after shift change but detected shortly before the official shift start would be evaluated as false negative. Likewise, the detection at the end of the latter shift would be evaluated as true positive, though it might be eight hours later. Consequently, to reach a more useful definition, *shift* was defined as +/- 4 hours of the correct starting time.
- Adding another case for evaluation: The classical contingency table was amended by a fifth and a sixth case to include aspects of correct timing of episodes. The sixth case was not defined explicitly in the study protocol and covers situations in which a SIRS episode was identified correctly but an additional false positive episode was detected within the same patient's stay (true positive and false positive).

**Berlin Heart:* Hetzer R, Loebe M, Potapov EV, et al. Circulatory support with pneumatic paracorporeal ventricular assist device in infants and children. *Ann. Thorac. Surg.* 1998;66(5):1498–506.

Appendix 3: Routine data used from patient data management system for CDSS assessment of all participants

**Routine data from PDMS**	**n**
Vital signs	
• Heart rate	2,684,005
• Respiratory rate	2,557,891
• Body temperature	1,149,370
Laboratory values	
• Leukocytes	2,889
• Neutrophils	86
Device-associated values	
• Controlled assisted ventilation	95,249
• Pacing	1,492
• Cooling	86

Appendix 4: Strategy for SIRS episode evaluation with examples

(A) **Strategy for preparing raw data for statistical analysis:**

<table>
<tr><th>Case</th><th>Ergebnis</th><th>CDSS</th><th>Goldstandard</th></tr>
<tr><td>(1)</td><td>False positive</td><td>>0 SIRS episode(s)</td><td>No SIRS episode</td></tr>
<tr><td>(2)</td><td>True positive</td><td>1. 1 SIRS episode +/-4h begin time
2. 2 SIRS episodes +/-4h begin time
*(and so on with
3,4,5 … x SIRS episodes)*</td><td>1. 1 SIRS episode
2. 2 SIRS episodes
*(and so on with
3,4,5 … x SIRS episodes)*</td></tr>
<tr><td colspan="4">The end of the SIRS episode is not taken into account. If the assessor documents additional SIRS episodes with less than 24 hours gap, these episodes will be treated as extension of the first episode and not evaluated as an additional “false positive”.</td></tr>
<tr><td>(3)</td><td>False negative</td><td>No SIRS episode</td><td>>0 SIRS episode(s)</td></tr>
<tr><td>(4)</td><td>True negative</td><td>No SIRS episode</td><td>No SIRS episode</td></tr>
<tr><td>(5)</td><td>False negative &
False positive</td><td>1. 1 SIRS episode starting 4h 01m after the begin time (or no episode at all) <u>and</u>
>0 additional SIRS episode(s)

2. 1 SIRS episode starting 4h 01m after the begin time (or no episode at all) <u>and</u>
>0 additional SIRS episode(s)

*(and so on with
3,4,5 … x SIRS episodes)*</td><td>1. 1 SIRS episode

2. 2 SIRS episodes

*(and so on with
3,4,5 … x SIRS episodes)*</td></tr>
</table>

(6)	False positive &<br>True positive	1. 1 SIRS episode starting at +/-4h of the begin time<br>and<br>>0 additional SIRS episode(s)<br>or<br>1. 1 SIRS episode starting 4h 01m before the start time but covering the complete reference standard episode<br>———<br>2. 2 SIRS episodes starting at +/-4h of the bgin time<br>and<br>>0 additional SIRS episode(s)<br>or<br>2. 2 SIRS episode(s) staring 4h 01m before the start time but covering the complete reference standard episode<br>*(and so on with 3,4,5 … x SIRS episodes)*	1. 1 SIRS episode<br>2. 2 SIRS episode(s)<br>*(and so on with 3,4,5 … x SIRS episodes)*

(B) **<u>Examples for preparing raw data for statistical analysis:</u>**

**Assessor**	**SIRS phase(s) during an intensive care unit stay**	**Result**
Reference standard		False positive
CDSS	*	

*(no SIRS episode in reference standard)

Reference standard		True positive
CDSS	*	

*(less than 24 hours between SIRS phases)

Reference standard		True positive
CDSS	*	

*(SIRS begin is correct, SIRS end is not evaluated)

Reference standard		False negative
CDSS	*	

*(no SIRS episode by assessor)

Reference standard		False negative
CDSS	*	

*(SIRS begin is too late)

Reference standard		True negative
CDSS	*	

*(no SIRS episode in both)

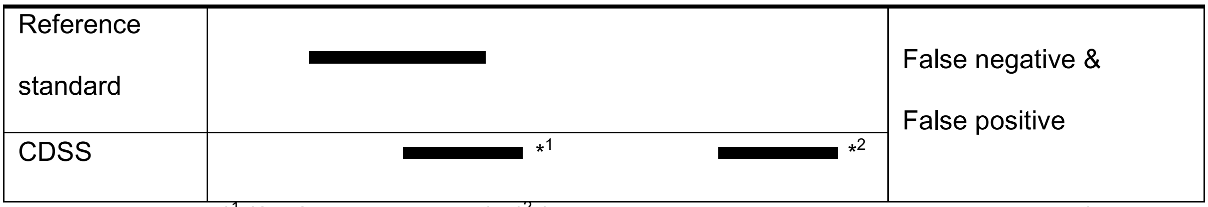

*[1] (SIRS begin is too late) *[2] (second episode is wrong, more than 24 hours later)

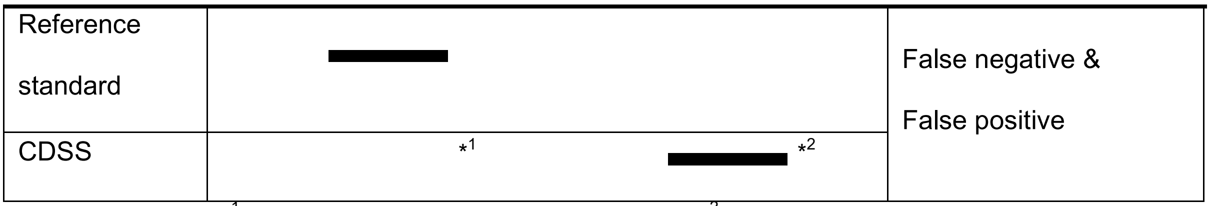

*[1] (No SIRS episode in reference standard) *[2] (no episode in reference standard)

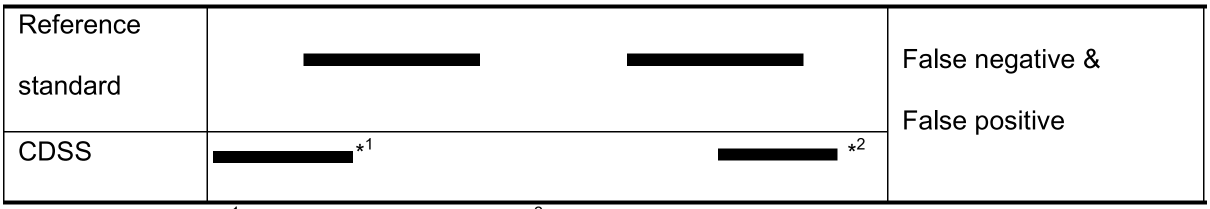

*[1] (SIRS begin is too early) *[2] (SIRS begin is too late)

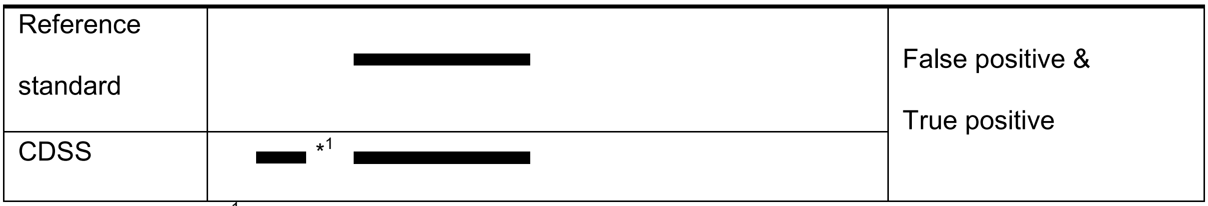

*[1] (SIRS begin is too early)

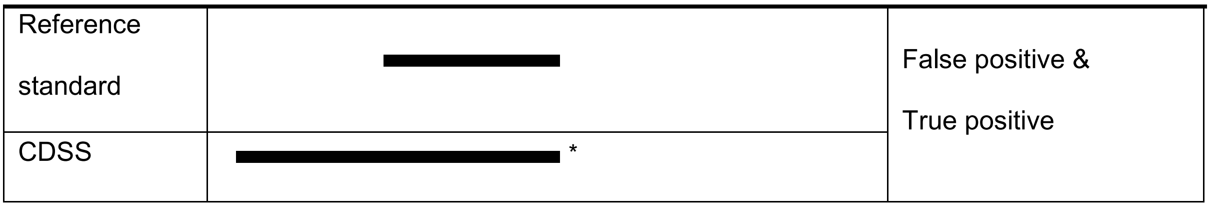

* (SIRS begin is too early but the complete episode is covered)

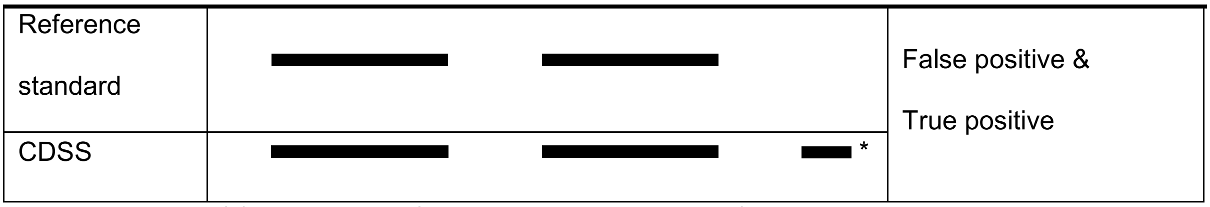

* (third episode is false, more than 24 hours later)

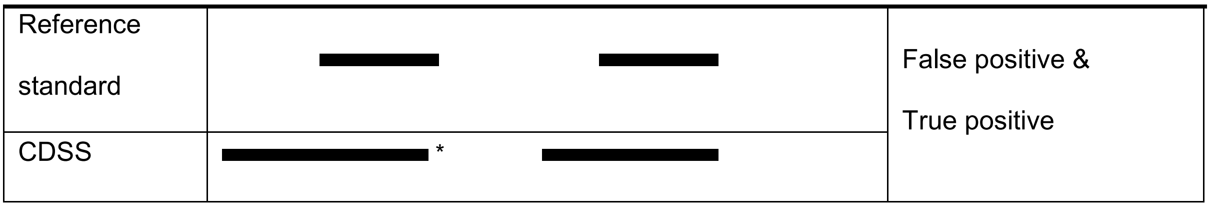

* (for both episodes: SIRS begin is too early, but the complete episode is covered)

Appendix 5: Flow diagram for recruited patients with intensive care days and patient's PICU stays

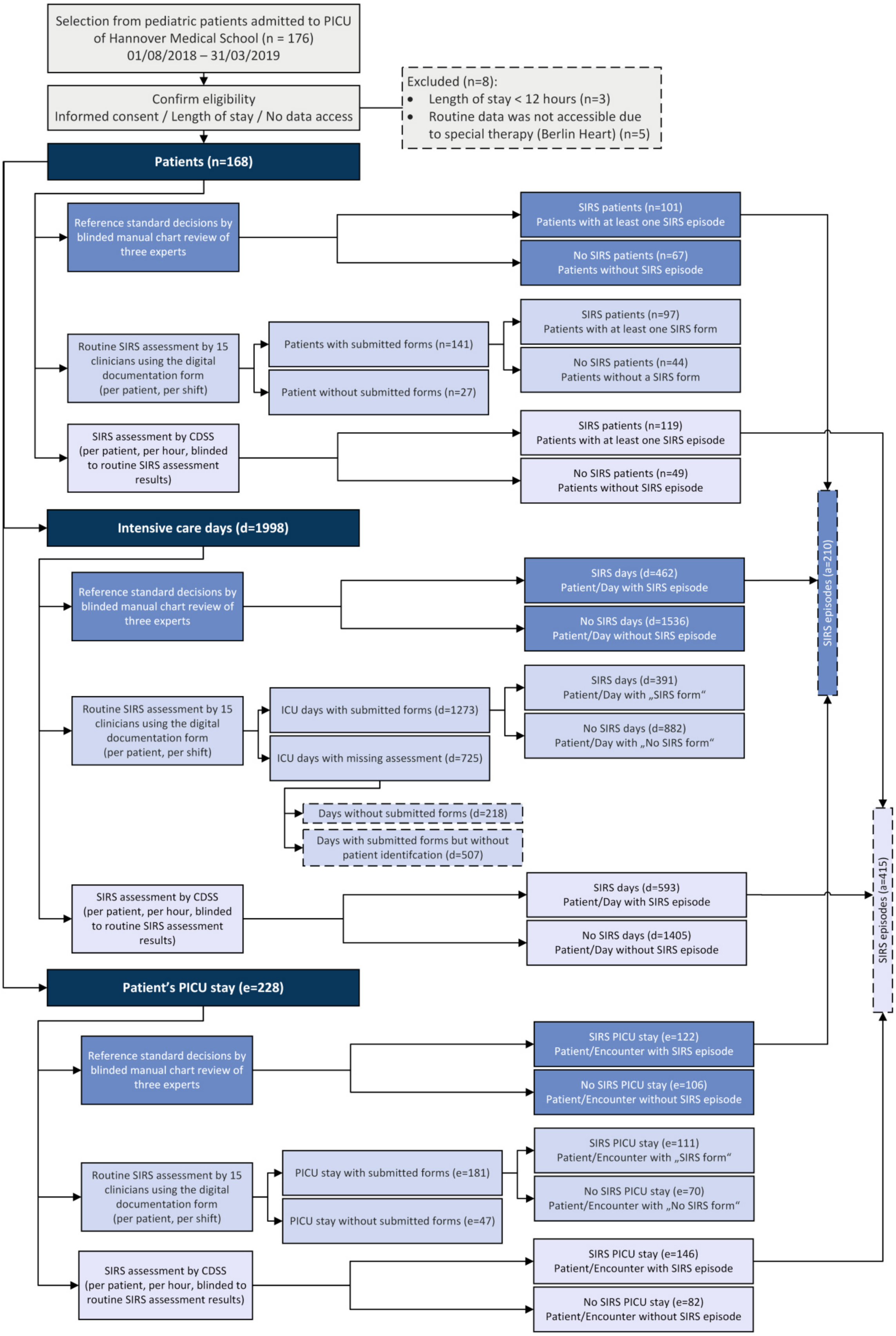

Appendix 6: Submitted forms during routine assessment per shift and per day

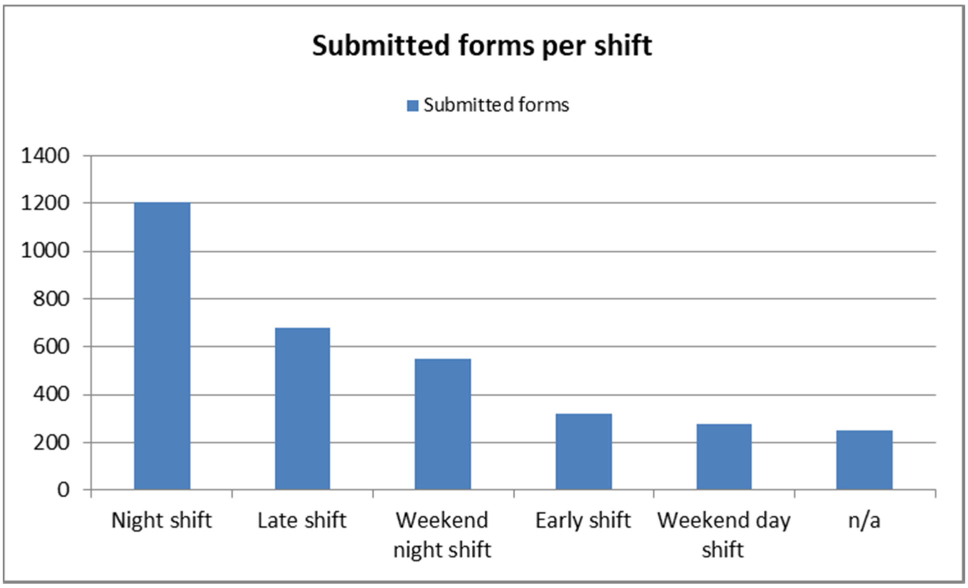

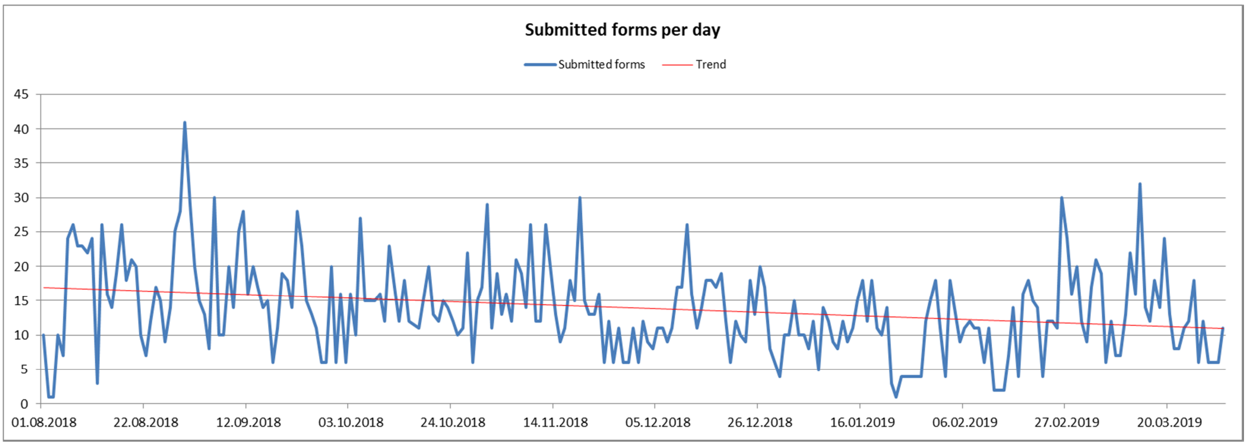

Appendix 7: False decisions from the CDSS diagnostic approach, classified into error categories

Category of error	Description	n
False negatives		**10**
Clinically different assessment/deviations from the ICCP guidelines	The implemented rules have triggered correctly and the database is also correct. However, clinical expertise has led to a different interpretation of the situation, so that the criteria that may apply do not apply after all (e.g. context factors exist that are not recorded by the system or certain correlations between parameters).	4
Poor data quality / wrong data base used	The data base of the parameter is not correct, i.e. the data from the source system may have been transferred incorrectly to the new data repository or data from before the recording on the ICU were used. The CDSS made the right decision, but did not get the right data / not enough data as input.	3
Body temperature		2
Heart rate		1
Incorrect rule triggering	The rule has triggered incorrectly/ did not work or the data was evaluated incorrectly (= not according to the implemented rules).	2
Respiratory rate (with mechanical ventilation)		2
Parameter’s under/exceedance is borderline	Basically the rule has been triggered correctly but the under/exceedance is borderline (e. g. only minimal).	1

	Experts did not assess this under/exceedance as clinically relevant.	
Body temperature		1
False positives		**143**
False positive days with 1 wrong parameter		*128*
False positive days with 2 wrong parameter		*15*
Underyling disease affecting parameter	There is an underlying disease. These are not yet recorded in CDSS.	24
Body temperature	*All errors were due to hypothermia*	12
Leuococytes		9
Heart rate		3
Parameter's under/exceedance is borderline	Basically the rule has been triggered correctly but the under/exceedance is borderline (e. g. only minimal). Experts did not assess this under/exceedance as clinically relevant.	92
Body temperature	*All errors were due to hypothermia*	63
Respiratory rate with mechanical ventilation		19
Leucocytes		2
Respiratory rate		5
Heart rate		3
Respiratory rate "Shivering"		3
Poor data quality / wrong data base used	The data base of the parameter is not correct, i.e. the data from the source system may have been	6

	transferred incorrectly to the new data repository or data from before the recording on the ICU may have been used. The CDSS made the right decision, but did not get the right data / not enough data as input.	
Leucocytes	*Data from before ICU recording*	4
Body temperature	*All errors were due to hypothermia*	1
Cooling device		1
Previous or parallel procedure	A previous or parallel procedure such as a surgery or MRI or extubating or ECMO changes the interpretation of the parameters. These correlations are currently not yet stored in the CDSS.	14
Hypothermia/Surgery		5
Hypothermia/ECMO		2
Leuocyctes/Surgery		1
Respiratory rate/Change of mechanical ventilation type or extubation		2
Hypothermia/MRI		1
Heart rate/ECMO		1
Heart rate/Transport		1
Respiratory rate/Surgery		1
Incorrect rule triggering	The rule has triggered incorrectly/ did not work or the data was evaluated incorrectly (= not according to the implemented rules).	6
Respiratory rate (with		6

mechanical ventilation)		
Medication	A medication was taken. These are not yet included in the CDSS, so that the utilization of the corresponding parameters cannot be adjusted.	4
Leucocytes		3
Respiratory rate		1
Clinically different assessment/deviations from the ICCP guidelines	The implemented rules have triggered correctly and the database is also correct. However, clinical expertise has led to a different interpretation of the situation, so that the criteria that may apply do not apply after all (e.g. context factors exist that are not recorded by the system or certain correlations between parameters).	9
**Additional, only for primary outcome measure on the level of patient**		
Incorrect start time	The SIRS criteria were detected correctly but episode was detected too late/too early (+/- 4 hours).	3
Too late	The patient’s stays were classified as false negative (case 3)	2
Too early	The patient’s stay was classified as false positive/true positive (case 6)	1
